# A perfusion protocol for lizards, including a method for brain removal

**DOI:** 10.1016/j.mex.2015.03.005

**Published:** 2015-03-12

**Authors:** Daniel Hoops

**Affiliations:** Evolution, Ecology & Genetics, Research School of Biology, The Australian National University, 116 Daley Road, Acton, ACT 2601, Australia

**Keywords:** Transcardial perfusion of lizards (with brain removal technique), Lizard, Reptile, Brain, Neuroscience, Perfusion

## Abstract

The goal of fixation is to rapidly and uniformly preserve tissue in a life-like state. Perfusion achieves optimal fixation by pumping fixative directly through an animal’s circulatory system. Standard perfusion techniques were developed primarily for application in mammals, which are traditional neuroscience research models. Increasingly, other vertebrate groups are also being used in neuroscience. Following mammalian perfusion protocols for non-mammalian vertebrates often results in failed perfusions. Here, I present a modified perfusion protocol suitable for lizards. Though geared towards standard brain perfusion, this protocol is easily modified for the perfusion of other tissues and for various specialized histological techniques.

• The two aortas of the lizard heart, emerging from a single ventricle, mean that care must be taken to place the perfusion needle in the correct aorta, unlike in mammals.
• Only the head and neck perfuse – the visceral organs will not decolour, and the body may not twitch.
• I also include a method for removing a lizard brain, which differs from mammals due to the incomplete and thicker skull of the lizard.

## Graphical abstract

**Figure:**
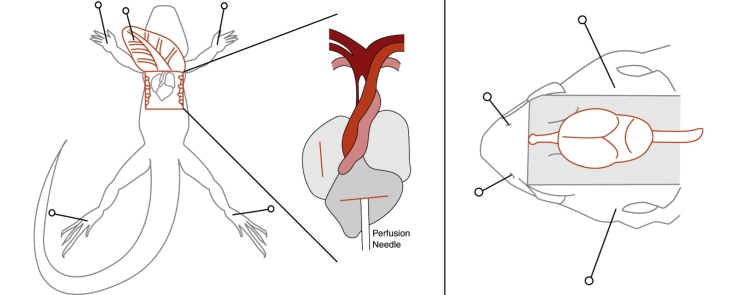

## Method details

Reliable fixation of nervous tissue is a prerequisite for valid histological investigations. Transcardial perfusion utilizes the circulatory system to distribute fixative throughout the organism quickly and efficiently. The animal, traditionally a mammal, is opened up just below the thoracic cavity, which is then entered through the diaphragm [1]. The heart is exposed, incisions are made into the right atrium and left ventricle, and a specialized, blunt-tipped needle (perfusion needle) is inserted into the left ventricle or the aorta. Fixative is then perfused throughout the animal. This has the added advantage of removing all blood, which may obscure histological features.

Recently, there have been significant advances in our understanding of the neurobiology of squamate reptiles (lizards and snakes), with particular emphasis on lizards [2], [3], [4]. Although perfusion is a commonly used method of fixation in lizard neuroscience, there is no published methodology for perfusing lizards. Lizards differ from mammals in anatomical and physiological ways that affect the perfusion method. Here, I have taken a standard mammalian perfusion protocol and modified it for lizards. This procedure has been tested on fifteen species of agamid and scincid lizards ranging between 3 g and 300 g. All materials necessary for this procedure are listed in Supplementary Materials 1.

**1. Prepare buffered solutions**

1.1 Prepare prefix perfusate, fixative perfusate and storage buffer (example solutions are listed in Supplementary Materials 2).

1.2 Prior to perfusion, warm buffers to room temperature. For mammal perfusions, solutions are often warmed to 37 °C (mammalian body temperature), however lizards are ectoterms and as such room temperature is their body temperature.

**2. Prepare apparatus**

Two types of devices are commonly used to perfuse liquids into the circulatory system: those depending on gravity to propel solutions, and those that use a pump system. I use a pump system, which is generally sufficient for protocols such as Nissl staining and immunohistochemistry. However for some purposes, such as electron microscopy, a gravity system may be more appropriate. Furthermore, gravity systems are useful in the field, as they do not require electricity.

2.1 Run distilled water through the perfusion tubing and perfusion needle to rinse.

2.2 Draw prefix perfusate into the perfusion tubing. 2 mL/5 g of lizard is sufficient to flush the blood from the circulatory system. Take care to avoid any bubbles forming in the tubing.

2.3 For a small lizard, the amount of prefix needed is so small that it is possible to store the entire amount in the perfusion tubing. In this case, measure out the required amount of prefix, draw it into the tubing, and mark off the appropriate place on the tubing with a permanent marker. Then continue to fill the tubing with fixative until there is no air left in the tubing. For larger lizards and when using a gravity system the switch from prefix to fixative has to occur during the perfusion, as with mammals. Always take care not to leave any air bubbles in the tubes.

2.4 Affix the perfusion needle to outlet end of the tubing.

2.5 Turn on the perfusion pump and adjust the flow rate until there is a weak but even flow of liquid out the end of the perfusion needle (no dripping). Flow rate is heavily dependant on the gauge of the needle and the model of perfusion pump. To find the correct pressure, start with a pressure at which liquid drips from the needle. Increase pressure slowly until the stream flows steadily. I use pressures between 75 and 100 mmHg. If a gravity system is being used instead of a pump system, an appropriate drip rate must be selected and maintained instead of an appropriate pressure.

**3. Anaesthesia**

3.1 Prepare a syringe with sodium pentobarbital, dose: 100 mg/kg [5]. I do not recommend inhalation anaesthetics, although I have seen them used. Lizards are able to hold their breath for long periods of time and so determining the dose of anaesthetic an animal has inhaled is difficult [5], [6].

3.2 Draw an equal volume of lignocaine into the syringe.

3.3 Administer the anaesthetic to the lizard via intraperitoneal injection. I recommend cooling the lizard to room temperature first by removing all heating elements from the lizard’s enclosure 12 h prior to perfusion. This makes injections easier as cooler lizards are calmer and have lower heart rates.

3.4 Complete anaesthesia occurs less than 10 min following an injection of anaesthetic. Once the lizard appears unconscious, test the level of anaesthesia by pinching the lizard’s toe sharply. If the animal is sufficiently anaesthetized, it will show no response.

**4. Surgery and perfusion**

I highly recommend using a dissection microscope for all of Sections 4 and 5. Relative to their body size, lizard hearts and brains are smaller than those of mammals, and some steps are quite intricate and delicate [7], [8].

4.1 Place the animal abdomen-up on the dissection pad.

**Fig. 1:**
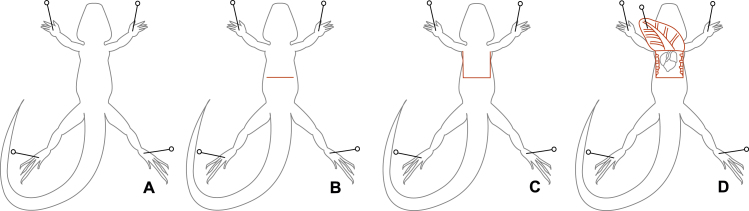
Proper preparation of an anaesthetized lizard for perfusion. (A) The lizard is pinned abdomen-up to a dissection pad (step 4.2). (B) A horizontal incision is made just below the sternum (step 4.3). (C) Two parallel incisions are made up from the horizontal incision to the clavicle (step 4.4). (D) the abdomen is lifted, inverted, and pinned to the dissection pad, exposing the heart (step 4.5).

4.2 Pin each foot to the dissection pad (Fig. 1A).

4.3 Open the abdomino-thoracic cavity, exposing the visceral organs. To do this, cut through the skin, abdominal wall, and peritoneal membrane just below the ribs, at the posterior end of the sternum (Fig. 1B).

4.4 There is no barrier between the visceral organs and the lungs (no diaphragm) [9], so immediately start cutting up the two sides of the chest, through skin, abdominal wall and ribs, to the level of the clavicle (Fig. 1C). As with all perfusions, while cutting through the ribs and body wall be careful not to sever any large blood vessels or damage organs such as the lungs, liver and intestines. A good way to do this is to orient the scissors up and away from the lizard while cutting. The pericardium may be attached to the body wall. If the heart does not detach easily, it can be gently pushed down using a small spatula.

4.5 Pin back the flap of skin (Fig. 1D). This should provide a clear view of the heart and major blood vessels.

4.6 In lizards the pericardium is stronger than in mammals and must be cut away manually. Carefully lift the pericardium away from the heart with forceps to create a tent of empty space and cut the pericardium open with very small scissors. Use two pairs of forceps to pull the pericardium and enlarge the hole, exposing the heart. Cut the heart free of the pericardium by cutting the *gubernaculum cordis*, which attaches at the base of the ventricle.

**Fig. 2:**
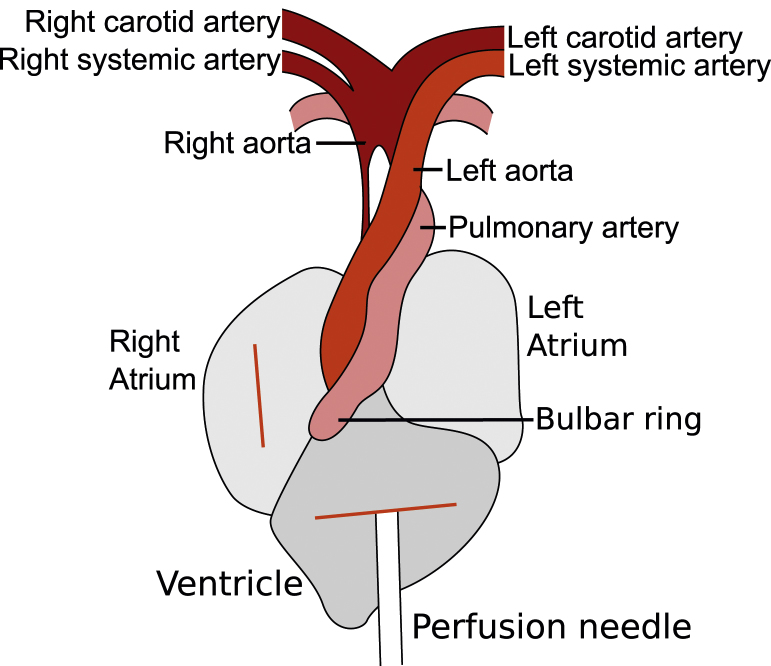
A schematic diagram of a lizard heart showing correct placement of the perfusion needle. Prior to needle insertion, incisions are made into the ventricle (step 4.7) and right atrium (step 4.8). The perfusion needle is then inserted though the incision in the ventricle into the right aorta (step 4.9). The tip of the perfusion needle is visible in the right aorta below the branch point of the right systemic artery.

4.7 Make a small incision in the posterior end of the ventricle (Fig. 2).

4.8 Make a small incision in the right atrium (Fig. 2).

4.9 Insert the perfusion needle through the incision in the ventricle into the correct aorta (Fig. 2). In mammals, the perfusion needle is inserted into the left ventricle or aorta. This is impossible in lizards because, unlike mammals, lizards have a single ventricle and two aortas (Fig. 2) [10], [11]. The two aortas and the pulmonary artery all emerge as a single trunk from a ventricular structure called the bulbar ring [10], [12]. For brain perfusion, the perfusion needle must be inserted into the right aorta, from which the carotid arteries emerge [10]. The base of the right aorta is obscured, so needle placement can be difficult (Fig. 2). When viewed under a dissection microscope, the tip of a correctly placed perfusion needle is visible through the transparent wall of the right aorta (Fig. 2). To perfuse tissues other than the brain, it may be appropriate to insert the perfusion needle into the left aorta or pulmonary artery.

It is important that there is no back flow of liquid out of the ventricle, so it is critical to choose a gauge of perfusion needle that fits securely into the aorta. In larger lizard species it may be possible to clamp the needle in the aorta.

4.10 Pin the heart in place. Carefully insert a pin through the ventricle, lateral to the perfusion needle and below the level of the bulbar ring. Push the pin down through the lizard’s dorsum and into the dissection pad. Avoid the spinal cord. Pins may also be used to stabilize the perfusion needle.

4.11 Turn on the perfusion pump, which should have been set to the correct pressure in step 2.5. Alternatively, if using a gravity system, open the valve.

4.12 Allow the perfusion to proceed until about 2.5 mL fixative per gram of lizard has been used.

4.13 Turn off the perfusion pump, remove the perfusion needle, and then remove the lizard from the dissection pad.

4.14 Remove the head posterior to the tympanic membranes (if visible) and the jaw bone.

**Fig. 3:**
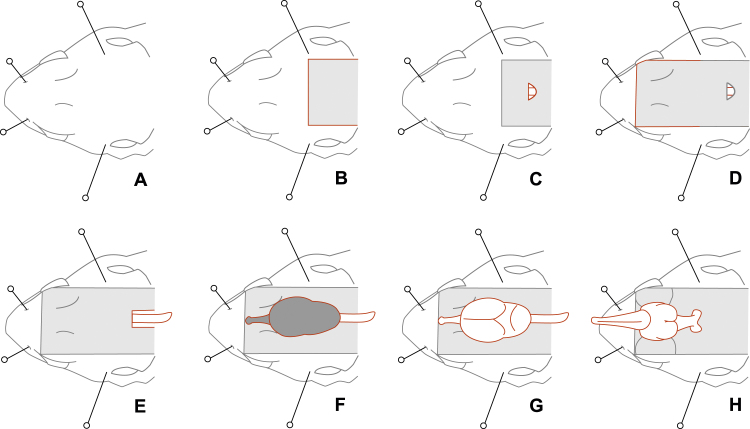
The process of removing a lizard brain. (A) The head is pinned to the dissection pad (step 4.15). The anterior pins are inserted through the nostrils, and the posterior pins through the upper temporal fenestras. (B) The posterior dorsal skin is removed, exposing the spinal column and base of the skull (step 4.16). (C) The membranes covering the brain are cut away from the gap between the skull and the spinal cord, exposing the brain (step 4.17). (D) The skin is removed from the dorsal surface of the head, including the eyes (step 5.2). (E) The spinal cord is exposed by removing the spine (step 5.4). (F) The skull is removed, exposing the dura-covered brain (steps 5.6–5.8). (G) The dura is removed (step 5.9). (H) The brain is carefully lifted and the ventral nerves severed (steps 5.12–5.13). The brain is now free of the head and can be removed.

**Fig. 4:**
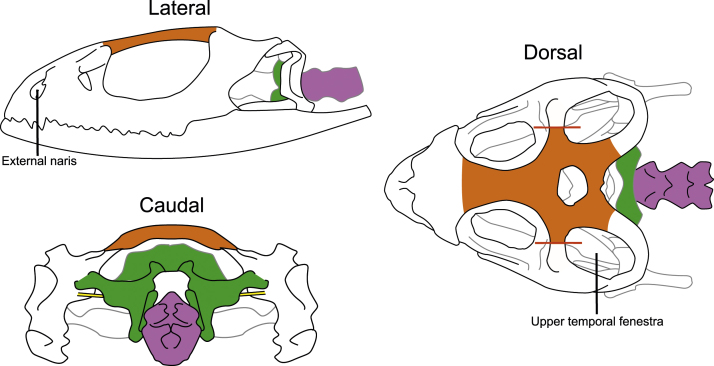
Lateral, dorsal, and caudal views of a standardized lizard skull which highlight the bones that must be removed to extract the brain. Prior to the removal of the skull, the head should be pinned through the external nares and upper temporal fenestras (step 5.1). First, the postorbital bone is cut, represented by the red lines (step 5.2). Second, the spine, shown in purple, is removed (step 5.4). Third, the posterior skull, in green, is removed (step 5.6). Fourth, the dorsal skull, in orange, is removed (steps 5.7–5.8). Finally, the ear bones, in yellow, are removed (step 5.11).

4.15 Pin the head to the dissection pad (Fig. 3A). Two pins are inserted through the external nares and two more pins through the upper temporal fenestras (Fig. 4).

4.16 Cut away the skin and muscle to expose the base of the spinal column (Fig. 3B).

4.17 Find the small opening at the base of the skull, where it attaches to the spine. In this space, cut away the membranes covering the brain, which are exposed in this gap (Fig. 3C).

4.18 Remove the head from the dissection pad and place the head in fixative for 24 h at 4 °C.

**5. Brain dissection**

5.1 Pin the head to the dissection pad. The lizard skull is far more flexible than the mammal skull and the brain is not fully encased in bone [13]. This means that pressure applied to the head of a lizard is more likely to result in damage to the brain. Pinning the head removes the risk of applying manual pressure to the head.

5.2 Cut the skin up both sides of the head (Fig. 3D). Cut through the postorbital bone (Fig. 4) and into the eyes.

5.3 Lift up the flap of skin and cut it away.

5.4 Pull the spine away from the spinal cord by grasping the spine firmly and pulling it straight backwards, away from the head (Fig. 3E and Fig. 4).

5.5 Pull the muscle away from the sides of the skull. There is no bone protecting the sides of the brain, so work carefully.

5.6 Carefully remove the posterior portion of the skull (Fig. 4).

5.7 Carefully remove the top portion of the skull (Fig. 4). The lizard skull is thicker than the mouse skull, and the brain is smaller relative to body size [8], [13]. To break apart and remove the skull I use a corneoscleral punch instead of the more traditional rongueurs (Supplementary Materials 1). I find the corneoscleral punch to be more adept at the delicate task of removing the thick skulls of lizards without damaging small brains. For very large lizard species, rongueurs may be useful.

5.8 Carefully remove the bone covering the olfactory tract and the olfactory bulbs (Fig. 4). While the brain sits just posterior to the eyes, the olfactory bulbs rest anterior to the eyes [14]. They are connected to the rest of the brain by a pair of fibre bundles known as the olfactory tracts, which are delicate and prone to tearing. Extreme care should be taken when removing the bone covering the olfactory tracts (the frontal bone) as the olfactory tracts may be attached to it. If the olfactory bulbs are not necessary for study, it is much more efficient to sever the olfactory tracts at their base and skip steps 5.8 and 5.10.

5.9 Cut away the dura (Fig. 3G) using the same method as for the pericardium in step 4.6. This membrane may be blackish (Fig. 3F) and is much stronger than in mammals.

5.10 Cut the olfactory bulbs away from the vomeronasal and olfactory nerves. Be careful to keep the olfactory tracts intact. This step can be skipped along with step 5.8 if the olfactory bulbs are not necessary for study.

5.11 Remove the ear bones by crushing them and then pulling them laterally away from the brain (Fig. 4).

5.12 Lift the brain carefully to expose the nerves attached to the brain, including the optic nerves.

5.13 Carefully sever the nerves, keeping the scissors well clear of the brain (Fig. 3H).

5.14 Grasp the spinal cord with tweezers and lift the brain from the lizard’s head.

**6. Post-fixation and storage**

6.1 Place the brain in fixative for 24 h at 4 °C.

6.2 Wash the brain in phosphate-buffered saline by replacing the solution three times, swirling gently between each exchange.

6.3 Store the brain in storage buffer at 4 °C. For sectioning, lizard brains can be paraffin-embedded and sectioned, cryosectioned, or vibratome-sectioned using methods identical to those used for mammalian brains [15]. If cryosectioning, sucrose in phosphate-buffered saline is an appropriate cryoprotectant for most protocols.

## Additional information

There are many important differences between mammals and reptiles that affect perfusion protocols, which I have outlined above. One additional difference between mammals and reptiles is that heart beat is maintained for longer after anaesthesia in reptiles compared to mammals, even once the heart is exposed (pers. obvs.). This allows more time to follow this procedure carefully and to make sure that the perfusion needle is inserted properly into the correct aorta.

**Fig. 5:**
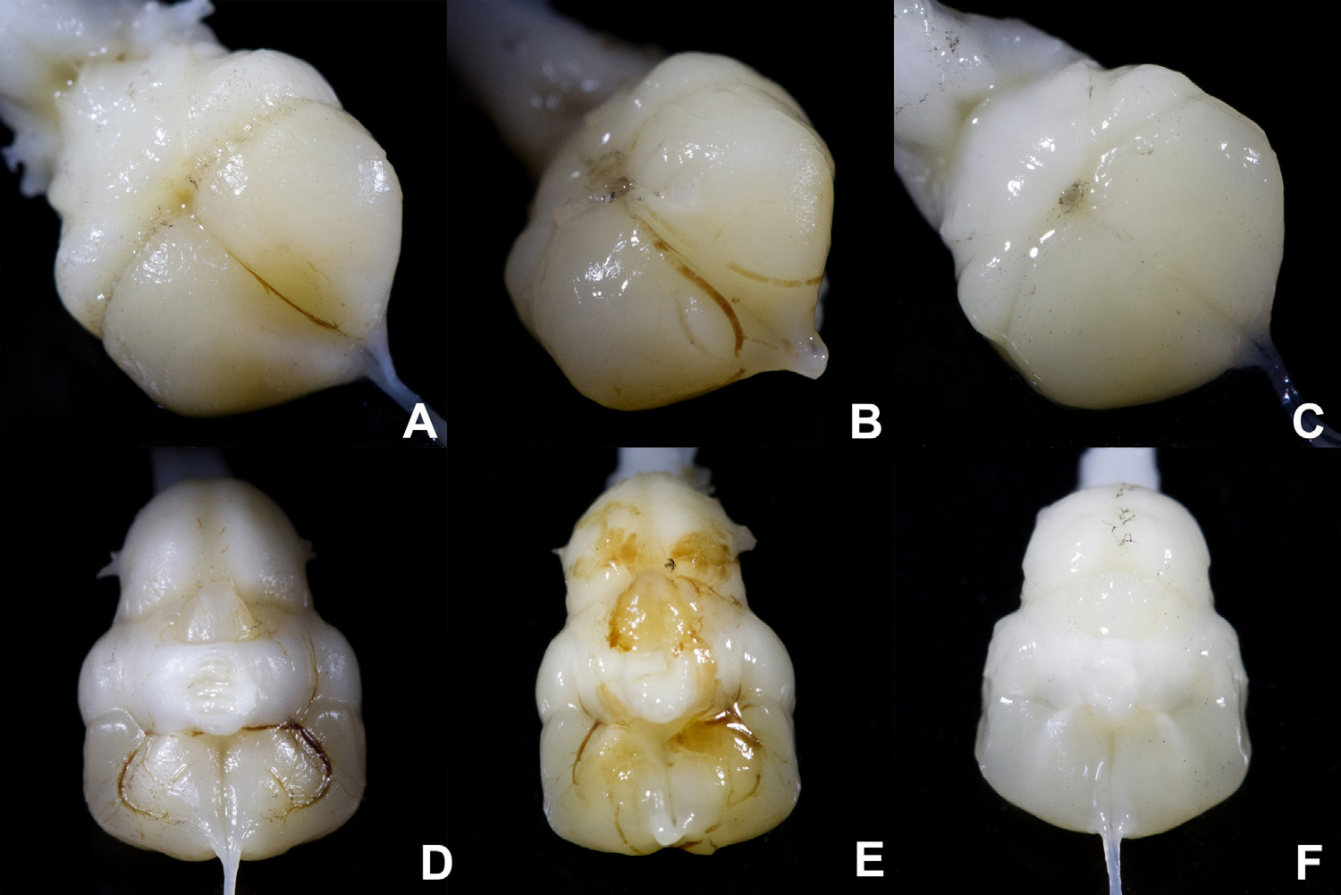
Dorsal (A–C) and ventral (D–F) views of successfully and unsuccessfully perfused brains. In unsuccessfully perfused brains (A, B, D and E), congealed blood is clearly visible in blood vessels on the surface of the brains, and the brains have an overall yellowish or pinkish tinge. A well-perfused brain (C and F) shows no visible blood vessels and the tissue is white.

With this protocol lizard brains can be successfully perfused, with complete blood clearance and full fixative penetrance. However, perfusions are delicate. Accidental cutting of a major blood vessel, slippage of the perfusion needle, air bubbles in the perfusion tubing and tachycardia prior to perfusion all may reduce the penetrance of fixative into the circulatory system and leave blood in the tissue [16]. As problems may occur without being noticed during the procedure, it is important to verify that the perfusion was successful. Once the brain has been extracted, it can be examined for any visibly red blood vessels along its surface (Fig. 5). Even if no blood vessels are visible, a yellowish or pinkish brain is the result of an unsuccessful perfusion, while a successful perfusion will result in a white brain (Fig. 5).

In mammals, two commonly used indicators of successful perfusion are loss of colour in the visceral organs, particularly the liver, due to blood clearing, and muscle-twitching when the fixative (paraformaldehyde) is initially pumped through the animal [1]. In lizards, the visceral organs do not perfuse and the liver will not decolour. This is because lizards have two aortas which circulate blood to the visceral organs (Fig. 2), but only the right aorta also circulates blood to the head, including the brain [10], [11]. A successful perfusion of the brain need only perfuse through the right aorta (Fig. 2) and does not fully perfuse the visceral organs. However, as the head is perfused, the mouth lining and tongue are white after a successful perfusion and pink after an unsuccessful one. Furthermore, for the same reason twitching may occur throughout the body, or may be restricted to the head region.

This is a basic protocol, suited for simple histological procedures such as Nissl staining, however it is easily adapted for different types of tissue analysis, such as immunohistochemistry and electron microscopy. Each procedure has its own specific modifications that would be made to the standard mammalian perfusion protocol [1], [15], those are also easily applied to this lizard perfusion protocol. For example, brains perfused using this method have been successfully imaged using magnetic resonance imaging (MRI), and the modifications to this protocol necessary for MRI are identical to those necessary for imaging mouse brains [17]. My goal is to make lizard neuroscience more accessible to researchers, and with this protocol I hope to help interested researchers study neuroscience in lizards.
