# Supplementary material for: A perfusion protocol for lizards, including a method for brain removal
Source: MethodsX. 2015 Mar 12;2:165–73. doi: 10.1016/j.mex.2015.03.005 (PMC4487709; doi:10.1016/j.mex.2015.03.005)
Supplement: Supplementary file 1 [file mmc1.docx]

**Table 1: Supplies needed for lizard perfusion, with suppliers.**

| **Name** | **Company** | **Catalog or Model Number** | **Steps** |
| --- | --- | --- | --- |
| **Consumables** | | | |
| Disodium Hydrogen Orthophosphate (Na_2_HPO_4_) | Ajax Finechem | 621 | 1.1 |
| Distilled Water |  |  | 1.1, 2.1 |
| Heparin | Hospira | 618963BAU | 1.1 |
| Hydrochloric Acid (HCl) | Merck | 109057 | 1.1 |
| Lignocaine | Troy Laboratories | CD0901 | 3.2 |
| Paraformaldehyde | Sigma-Aldrich | P6148 | 1.1 |
| Pentobarbitone | Troy Laboratories | CD0961 | 3.1 |
| Potassium Chloride (KCl) | Merck | 104936 | 1.1 |
| Potassium Dihydrogen Orthophosphate (KH_2_PO_4_) | Chem-Supply | PA009 | 1.1 |
| Sodium Azide (NaN_3_) | Merck | 822335 | 1.1 |
| Sodium Chloride (NaCl) | Merck | 106404 | 1.1 |
| Sodium Hydroxide (NaOH) | Merck | 106469 | 1.1 |
| **Equipment** | | | |
| Coarse Insect Pins | Fine Science | 26000-55 | 4.2, 4.5, 4.10, 4.15, 5.1 |
| Corneoscleral Punch | World Precision Instruments | 500143 | 5.6-5.8 |
| Dissecting Scissors | World Precision Instruments | 14393 | 4.3-4.4, 4.16, 5.2-5.3 |
| Dissection Microscope^1^ | Leica Microsystems | Leica S4 E | All of 4 & 5 |
| Dissection Pad | Southern Biological | G3.17 | 4.1-4.2, 4.15, 5.1 |
| Household Scissors, 20 cm | Celco | 230200 | 4.14 |
| Micro-spatula | Pacific Laboratory Products | 2620/130X4 | 4.4, 5.12-5.13 |
| Needle Tip, 25 Gauge | Terumo | SG3-2516 | 3.1-3.3 |
| Perfusion Needle^2^ | Leica Biosystems | 39471024 | 2.4-2.5, 4.9 |
| Perusion Pump | Gilson | Minipuls II | 2.1-2.5, 4.11 |
| Syringe, 1 mL | BD | 302100 | 3.1-3.3 |
| Tweezers | ProSciTech | T05-811 | 4.3-4.6, 4.9, 4.17, 5.3-5.5, 5.9, 5.11, 5.14 |
| Vannas Scissors | World Precision Instruments | 500086 | 4.6-4.8, 4.17, 5.9-5.10, 5.13 |
| Ventilated Dissection Table |  |  | All of 4 & 5 |
| Vials for lizard brains |  |  | 6.1-6.3 |
| Vials for lizard heads |  |  | 4.18 |

^1^The dissection microscope I use was manufactured by Wild Heerburgg, which is now part of Leica. A currently available microscope equivalent to the one I use is listed.

^2^I made my own perfusion needle from a glass pipette pulled to fit the lizard's aorta and glued over a butterfly needle. Depending on the size of the lizard's aorta, a commercial perfusion needle may suffice, one may need to be made.
