# Supplementary material for: A perfusion protocol for lizards, including a method for brain removal
Source: MethodsX. 2015 Mar 12;2:165–73. doi: 10.1016/j.mex.2015.03.005 (PMC4487709; doi:10.1016/j.mex.2015.03.005)
Supplement: Supplementary file 2 [file mmc2.docx]

**Supplementary Materials 2: Solution Recipes**

**Stock Solution: 10x Phosphate Buffered Saline (PBS)**

80.0 g NaCl

2.0 g KCl

14.4 g Na_2_HPO_4_

2.4 g KH_2_PO_4_

Distilled Water to 1000 mL

1. Dissolve all salts into distilled water.

2. Store at room temperature.

**Prefix Perfusate: Heparinised PBS**

Phosphate buffered saline with 1.0% heparin is used to flush the blood from the lizard prior to fixative perfusion.

100 mL 10x PBS

10 units Heparin

Distilled Water to 1000 mL

1. Add heparin to 10x PBS, then add water to 1000 mL.

2. Check pH. If necessary, add NaOH or HCl until the solution has a pH of 7.4.

3. Store at 4**°**C.

**Fixative Perfusate: 4% Paraformaldehyde (PFA) in PBS**

4% PFA is used to fix the tissue and for post-fixation. Preparation and use should always been in a well-ventilated area, such as under a fume hood or over a ventilated dissection table.

40.0 g Paraformaldehyde

100mL 10x PBS

Distilled water to 800 mL

NaOH pellets (or 10 M NaOH)

Additional distilled water to 1000 mL

1. Add paraformaldehyde to 10x PBS.

2. Add distilled water to 800 mL.

3. Stir vigorously and heat to 60-65**°**C. Do not allow the solution to heat above 65**°**C, this will denature the paraformaldehyde and ruin the solution.

4. Add three pellets of NaOH and stir for 30 minutes

5. If the solution has not cleared, add two additional pellets of NaOH and stir for an additional 15 minutes. Repeat until the solution has cleared.

6. Add distilled water to 1000 mL

7. Adjust pH to 7.4 using HCl.

8. Filter.

9. Store at 4**°**C for up to one week.

**Storage Buffer: PBS with Sodium Azide**

Phosphate-buffered saline with sodium azide is used to prevent any bacteria or fungal growth during long-term storage. I have successfully stained brains with Nissl stain and fluoronissl stain after storing them in this solution for over one year. However, this is unlikely to work for more advanced protocols.

100 mL 10x PBS

0.090 g NaN_3_

Distilled Water to 1000 mL

1. Add NaN_3_ to 10x PBS, then add water to 1000 mL.

2. Stir until dissolved.

2. Adjust pH to 7.4 with NaOH and HCl.

3. Store at 4**°**C.
